# Co-existence of Ovarian Teratomas With Other Gynecological Tumors

**DOI:** 10.7759/cureus.58068

**Published:** 2024-04-11

**Authors:** Michail Matalliotakis, Charoula Matalliotaki, Ioannis Tsakiridis, Themistoklis Dagklis, Georgios Michos, Andreas Romanos, Konstantinos Krithinakis, Ioannis A Kalogiannidis

**Affiliations:** 1 Obstetrics and Gynecology, Venizeleio General Hospital, Heraklion, GRC; 2 Third Department of Obstetrics and Gynecology, Aristotle University of Thessaloniki, Thessaloniki, GRC

**Keywords:** cancer, benign gynecological disease, ovarian mass, immature teratoma, benign mature cystic teratoma

## Abstract

Introduction: This study aims to investigate the co-existence of ovarian teratomas with other benign or malignant gynecological tumors in women who underwent gynecological surgery.

Methods: We retrospectively reviewed all women who underwent gynecological surgery over a 15-year period. Pre-operative, surgical, and histological records were obtained from women who presented with gynecological pathology, aiming to discover a possible link between ovarian teratomas and other gynecological tumors.

Results: Of the total patient sample, 288 (8.2%) had a mature teratoma, and 9 (0.3%) had an immature teratoma. The mean age was 38.0±13.3 years and 30.9±11.1 years, respectively. Women with mature teratoma showed a positive correlation with struma ovarii (SO, p=0.001). Moreover, we reported a positive linear relationship between struma ovarri and thecoma. Of the 288 women with a mature teratoma, 1 (0.3%) had co-existent endometrioid ovarian cancer, and 1 (0.3%) had borderline cancer. There were 14 women (4.9%) with a co-existent serous cystadenoma, 7 (2.4%) with a mucin cystadenoma, 1 (0.3%) with a thecoma, 4 (1.4%) with struma ovarii, 3 (1.0%) had Brenner cyst, 3 (1.0%) had ovarian fibroma, 2 had endometriosis (0.7%), and 8 (2.8%) had endometriomas. Of a total of nine women with immature teratomas, one (11.1%) had a serous cystadenoma.

Conclusions: Ovarian teratomas may co-exist with other gynecological diseases. Our study reports various cases of the co-existence of several gynecological tumors with teratomas.

## Introduction

Ovarian teratomas are germ cell tumors (GCTs) of the ovaries that are characterized by three subtypes, including mature teratoma (dermoid cyst), immature teratoma, and monodermal teratoma.

Mature teratoma constitutes the most common subtype of GCTs, with an incidence of about 1.2-14.2 cases per 100,000 people yearly. It accounts for about 95% of teratomas and 10% of all ovarian tumors. Even though they are benign, rarely in older women, there is a risk of malignant transformation (between 0.5% and 3%) [1]. Immature teratoma, mostly seen in younger ages, represents a rare entity and accounts for less than 1% of ovarian cancers. Due to its rarity, the incidence varies, but it signifies 10% of GCTs, 36% of GCTs in young adults, and about half of malignant GCTs [2]. The least frequent subtype, monodermal ovarian teratoma, includes mainly struma ovarii (SO) that are benign, although there is a risk of 5-10% for malignancy [3].

Clinically, patients with ovarian teratomas can be asymptomatic or manifest pelvic pain or discomfort upon examination, which may present as a complication due to torsion, rupture, or a complicated peritoneal infection. These overlapping symptoms can also be a manifestation of other concomitant gynecologic conditions that can be detected by sonographic imaging, CT scan, or MRI; however, the diagnosis is confirmed by histology [1,2,4,5,6,7].

The purpose of our study was to evaluate a possible clinically important coexistence between mature and immature ovarian teratomas and other benign and malignant gynecological conditions in a large number of women who underwent gynecological surgery.

## Materials and methods

This retrospective study was carried out at the Department of Obstetrics and Gynecology at Venizeleio General Hospital of Heraklion, Crete, and the Third Department of Obstetrics and Gynecology at Aristotle University of Thessaloniki. All women who underwent gynecologic surgery from 2005 to 2020, such as total abdominal hysterectomy (TAH) with or without unilateral (USO) or bilateral salpingo-oophorectomy (BSO), hysteroscopy, and/or dilation and curettage, were eligible to participate in the study.

Clinical, preoperative, surgical, and pathological records were obtained from 3490 women who presented with one or more gynecological conditions. The aim was to discover a possible link between mature and immature teratomas and other gynecological pathologies. Gynecological conditions included cysts, cystadenomas, endometriomas, endometriosis, fibroids, and ovarian malignancies.

All demographic and histological data were retrieved from the medical records. We excluded cases with inadequate medical records and those lacking histological diagnoses. The Ethics Committee of both departments approved the study protocol (no. 124/17/2019, no. 94/23-4-20).

Statistical analysis

Statistical analyses were performed using SPSS software version 25 (SPSS, Inc., Chicago, USA). Descriptive statistics were used to calculate the mean, average, and standard deviation of all the data. Values were considered statistically significant at p<0.05. The results are reported as mean ± SD or as percentages, where appropriate. Graphs were generated using GraphPad Prism (GraphPad Software, La Jolla, CA).

## Results

**Figure 1:**
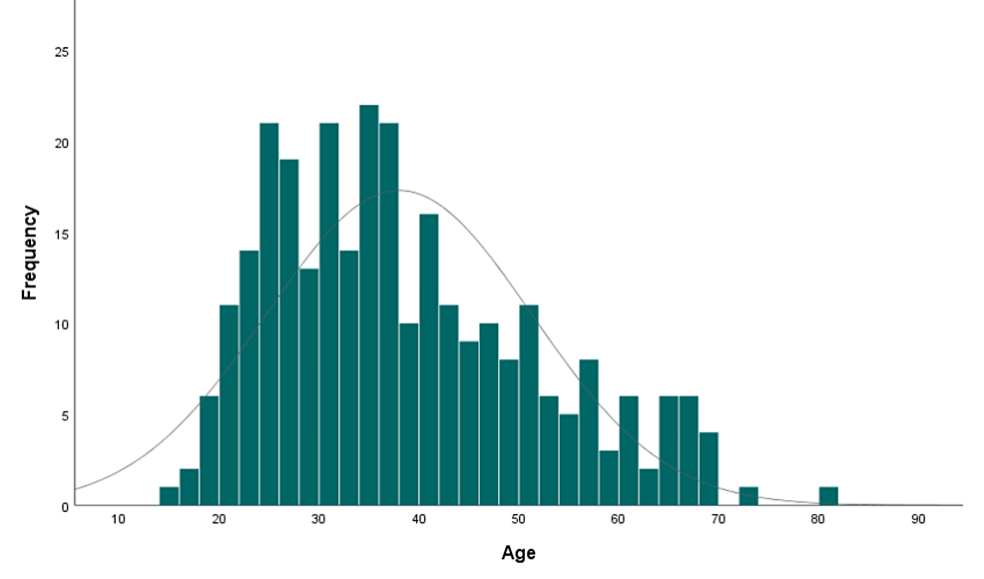
Age distribution in women with mature teratoma.

Of a total series of 3490 cases, 288 (8.20%) had mature teratoma, and 9 (0.30%) had immature teratoma, with a mean age of 38.0±13.3 years (range 15-81 years; Figure 1) and 30.9±11.1 years (range 17-54 years), respectively.

**Figure 2:**
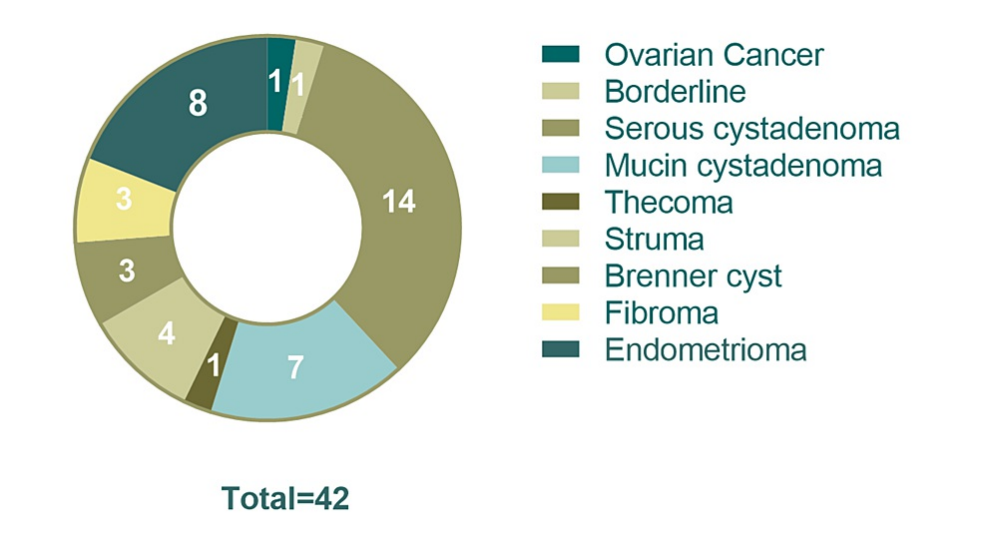
Most common gynecological conditions detected in the whole sample of teratomas. Total = 42. The total number of gynecological conditions co-existed with teratomas.

**Figure 3:**
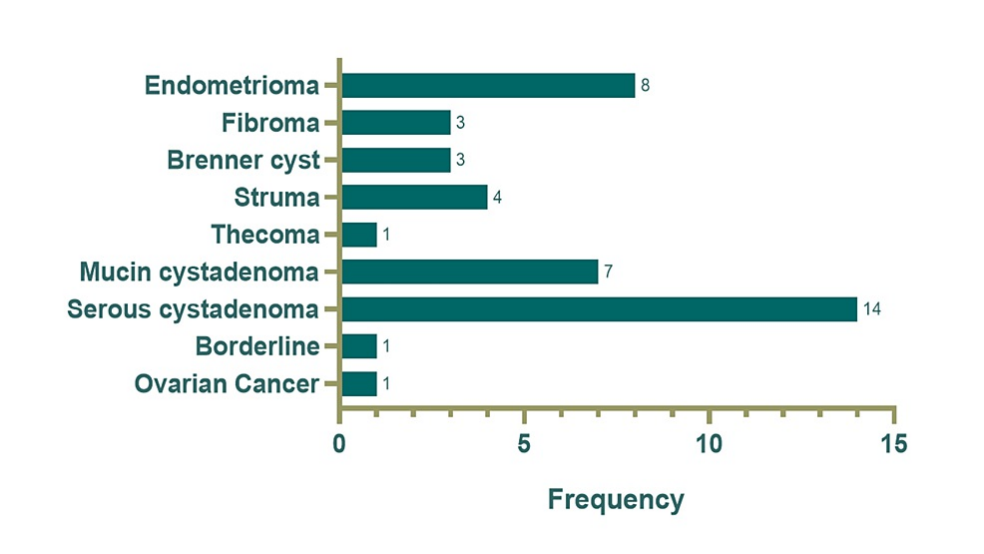
Coexistence of teratomas with various benign, borderline, and malignant ovarian masses.

Of the 288 cases with mature teratoma, 11 (3.8%) were isolated findings, whereas in 96.2%, there was additional gynecological pathology. Of the 288 women with mature teratoma, 1 had ovarian cancer (0.3%), specifically endometrioid, 1 (0.3%) had borderline cancer, 14 women (4.9%) had serous cystadenoma, 7 (2.4%) had mucin cystadenoma, 1 (0.3%) had thecoma, 4 (1.4%) had struma ovarii, 3 (1.0%) had Brenner cyst, 3 (1.0%) had ovarian fibroma, 2 had endometriosis (0.7%), and 8 (2.8%) had endometriomas. Of a total of nine women with immature teratomas, one (11.1%) had serous cystadenoma (Figures 2-3).

Women with mature teratomas showed a positive correlation with struma ovarii (p=0.001). A negative linear relationship was noticed with the presence of serous cystadenoma (p=0.002), paraovarian cyst (p=0.001), morgani cyst (p=0.010), and endometrioma (p<0.001). A negative correlation was also noticed regarding cervical and endometrial cancer (p<0.001), endometrial hyperplasia with and without atypia (p=0.013 and p=0.007, respectively), fibroids (p<0.001), endometriosis (p=0.002), and adenomyosis (p<0.001). No correlation was noticed with either ovarian cancer (p=0.106) or ovarian tumors of borderline malignancy (p=0.463). Immature teratomas did not show a significant correlation coefficient. Upon further examination of the data regarding struma ovarii, in the total sample of 3490 women, 12 cases were identified with a mean age of 50.4±17.7 years (range 26-69 years). Pearson’s correlation coefficient revealed a positive linear relationship with thecoma (p=0.001).

## Discussion

Although various reports have been published on ovarian teratomas, this large study investigated a possible noteworthy co-existence between 297 cases of ovarian teratomas and several benign, premalignant, or malignant gynecological diseases in women who have undergone surgery due to a gynecological condition.

Even though various theories have been linked to the pathogenesis of teratomas, the germ cell theory is widely accepted due to the anatomic distribution of the tumors and the fact that the tumor occurs during the reproductive years. Although the exact pathogenesis of ovarian teratomas is still obscure, they are believed to develop due to faulty meiosis type II in totipotent germ cells. Rarely, triploidy, trisomy, and mosaicism have been detected in ovarian teratomas; however, most of them have a normal 46, XX karyotype [3].

Mature teratomas present mostly as unilocular cysts, localized unilaterally with a variable size, characterized by a well-defined capsule. They can originate from ectoderm, mesoderm, or endodermal layers and can comprise hair, skin, sebaceous substances, and neural tissue. According to Peterson et al. and Whitecar et al., they appear mainly in the reproductive years and account for 30% of adnexal masses diagnosed during pregnancy [8,9].

Immature teratomas tend to be moderately larger than mature teratomas and are typically detected in adolescence. They are mostly solid with a non-well-defined capsule and differ from mature teratomas in the presence of embryonic elements, frequently primitive neuroepithelium. They are histologically graded depending on the neural elements, and this grading indicates a prognostic survival factor [3].

Teratomas represent around 11% of all ovarian masses in the literature, with mature teratomas accounting for 95% of all teratomas [1]. Our findings are similar, with teratomas accounting for 8.60% of all ovarian masses and mature teratomas for 96.9% of all teratomas. Ovarian teratomas are usually present in adolescent and premenarchal girls or are incidentally diagnosed later in life during imaging for other indications, with similar age groups in appearance for both mature and immature teratomas [10,11]. In our study, mature teratomas showed a mean age of 38.0±13.3 years and immature teratomas 30.9±11.1 years. This trend towards the higher age margin can be explained by the study design, given that the participants were women undergoing a surgical procedure that has specific indications when concerning children or adolescent patients [11,12].

Regarding the co-existence of mature teratomas and other benign gynecological conditions, few case reports of collision tumors exist in the literature. Pearson’s analysis of our sample corroborates this since it revealed a negative linear relationship with most tumors. The most reported entity appears to be mucin cystadenoma, with seven cases [13-16]. Of our patients, seven had mucin cystadenoma and mature teratoma. Serous cystadenoma has also previously been reported in five cases [13,17-20]. Our data showed that 14 women had serous cystadenoma and mature teratoma, while 1 woman had immature teratoma.

Further research is required to explore the pathway through which ovarian teratomas are negatively associated with a number of benign and malignant gynecological pathologies. An interesting example of this would be a previously confirmed left lateral anatomical distribution of endometriomas versus a right lateral anatomical distribution of ovarian teratomas [5]. Endometriosis is reported in six previously published articles with mature teratomas [21-26]. In our case, two women with mature teratomas also had endometriosis, and eight women had endometriomas.

Thecoma is the next most reported tumor, with three cases described by Morimitsu et al. and Emeka et al. [27,28]. One woman, in our case, had thecoma and mature teratoma. Also, Borges et al. report the co-existence of a Brenner tumor, which was found in three women in our study [14].

As far as the negative correlation of teratomas with fibroids is concerned, according to the literature, fibroids are fueled by gene mutations, whereas ovarian teratomas evolve through changes in gene expression; thus, these two pathologies present distinct entities [29].

Accounting for the presence of malignant tumors with mature teratomas, 10 cases of ovarian carcinoma are reported, one case of adenomatoid tumor, and one case of fibrothecoma, which are occasionally malignant [13,30-40]. Of our patients, one had endometrioid cancer, and one had borderline cancer.

Monodermal teratomas are well-differentiated unilateral neoplasms and have a predominance of a single tissue type that gives rise to a struma ovarii tumor, an ovarian carcinoid tumor, or an ovarian teratoma with neural differentiation [3].

Struma ovarii represents the most common type of monodermal teratoma. Upon macroscopical examination, the tumor appears solid, solid-cystic, or cystic with a solid component. It appears mainly during perimenopause and comprises less than 1% of all ovarian tumors, 2% of GCTs, and about 3% of dermoid tumors. It appears as a unilateral mass of various sizes [41,42].

Well, of note, in 1889, Boetlin, in 1895 von Kahlden, and Gottschalk, almost 5 years later, were the first authors who described SO as a rare ovarian mass that is histologically composed of thyroid tissue originating from the ovarian follicles. SO is also called ovarian goiter since the proportion of thyroid tissue occupies more than half of the specimen, compared to mature cystic teratomas, where the thyroid tissue involved is less than 15%. SOs are mostly benign, with a risk of malignant transformation of less than 5% [43]. Usually, asymptomatic SO is found incidentally on the surgical specimen of a teratoma or upon clinical examination and/or sonographic evaluation. Occasionally, larger masses may be complicated with ascites, with or without pleural effusion (Pseudo-Meigs syndrome), which disappears after the operation. Rarely, a hormonally active SO is associated with thyrotoxicosis [41-43]. There are few case reports that report the coexistence of mature teratomas and SO [44,45]. In our series, out of 288 cases with mature teratoma, we confirmed the co-existence of mature teratoma and SO in four specimens. The results of Pearson’s correlation coefficient showed a positive correlation between women with mature teratoma and SO (p=0.001). Recently, in 2019, we investigated retrospectively the association between teratomas and endometriomas. In that study, we detected 14 cases of SO out of 172 women with dermoid cysts [5]. Moreover, according to the current literature, a small number of case reports and case series confirm the co-existence of SO with other ovarian masses, such as mucinous or serous cystadenoma, Brenner tumor, thecoma, and ovarian fibroma [46-50]. In our total sample of 3490 women, we detected 12 cases of SO. Pearson’s correlation coefficient revealed a positive linear relationship with thecoma (p=0.001).

In all ovarian teratomas, the optimal follow-up regime after excision will result in early evaluation of recurrence or malignant transformation. Harada et al. retrospectively indicated that younger age, larger cysts, and bilateral occurrence of mature teratomas are prognostic factors for recurrence [51].

Chiang et al. concluded that long-term follow-up is suggested for mature teratomas with a diagnosis of a larger tumor or those with a tumor containing a solid component since there is a risk of malignant transformation to squamous cell carcinoma [52].

The retrospective nature of the study and the lack of follow-up denote its limitations. However, the large number of specimens and the histological confirmation of all cases present the strengths of our work.

## Conclusions

Teratomas, although rare, may co-exist with other ovarian tumors. Such a scenario poses a challenge to clinicians due to the difficulty of distinguishing them solely by imaging. This should raise awareness so that the therapeutic approach is based on the masses with a higher risk of malignancy. Long-term follow-up of ovarian teratoma cases would help to validate and reinforce the research results. To the best of our knowledge, the present study is the first to report a significant number of cases of co-existence of various gynecological tumors with teratomas, almost doubling the number of existing reports in the literature.
